# Supplementary material for: Intake of Low Glycaemic Index Foods but Not Probiotics Is Associated with Atherosclerosis Risk in Women with Polycystic Ovary Syndrome
Source: Life (Basel). 2023 Mar 15;13(3):799. doi: 10.3390/life13030799 (PMC10052525; doi:10.3390/life13030799)
Supplement: Supplementary file 1 [file life-13-00799-s001.zip › life-2220137-supplementary.pdf]

Supplement Table S1: Anthropometric measurements characteristics of the study group.

Characteristics	Low AIP		High AIP		*P*
	n	%	n	%	
BMI					
< 25 kg/m^2 normal	49	72	21	36	< 0.00*
≥ 25 kg/m^2 overweight and obese	19	28	38	64	
Waist circumference					
< 80 cm low risk	43	63	19	32	< 0.00*
≥ 80 cm at risk	25	37	40	68	
WHR (-)					
< 0.85 gynoid	55	80	36	61	0.04*
≥ 0.85 android	13	20	23	39	

*p< 0.05.Values were calculated with the chi2 test.

Supplement Table S2: Odds ratios (O.R.s with 95% confidence interval (95% CI)) of the high atherogenic index of plasma according to the diet quality indexes.

Food indexes	Atherogenic Index of Plasma ≥ 0.11		
	Occurrence (%)/N	Crude OR (CI 95%)	OR adjusted for BMI and age (CI 95%)
ProDI-4 probiotic diet index[1] ≥ upper quartile	(16.54)/21	1.08 (0.51; 2.26); p = 0.83	1.10 (0.49; 2.49); p = 0.81
LGIDI-4 low glycaemic diet index[2] ≥ upper quartile	(26.77)/10	0.37 (0.16; 0.88); p = 0.02*	0.32 (0.12; 0.85); p = 0.02*
pHDI-10[3] pro-healthy diet index ≥ upper quartile	(8.66)/11	0.51 (0.22; 1.19); p = 0.11	0.43 (0.17; 1.13); p = 0.08
nHDI-14[4] non-healthy diet index ≥ upper quartile	(10.24)/13	0.78 (0.34; 1.79); p = 0.56	1.39 (0.55; 3.52); p = 0.48

[1] probiotic food group: Fermented milk drinks, Tinned vegetables, Fresh cheese curd products, cheese; [2] low glycaemic food groups: wholemeal bread, buckwheat, oats, whole-grain pasta or other coarse-ground groats, legumes-based foods, and vegetables; [3] pro-healthy dietary index [34]; [4]non-healthy dietary index [34]; The *p* values below the threshold of statistical significance are marked with the *p< 0.05

T
